# Dengue Cardiomyopathy Treatment Strategy Optimisation by Integrating Point of Care Ultrasound (POCUS) with VExUS

**DOI:** 10.24908/pocus.v9i2.17706

**Published:** 2024-11-15

**Authors:** Wei Ven Chin, Mae Jane Khaw

**Affiliations:** 1 Acute Internal Medicine, Sarawak General Hospital MYS, Sarawak; 2 Medical Department, Limbang Hospital Sarawak MYS

**Keywords:** Case Report, Dengue Fever, Dengue Cardiomyopathy, Point-of-Care Ultrasound, POCUS, Bedside Ultrasound, VExUS

## Abstract

Dengue fever is an arthropod-borne viral disease that is widespread throughout the world. We report a case of dengue cardiomyopathy that was detected and treated to best effect using point of care ultrasound (POCUS) and the VExUS score. A 75-year-old lady with no known comorbidities presented with a ten-day history of fever, vomiting, loose stool, and poor appetite. Upon arrival, she was febrile and hypotensive. POCUS examination showed reduced left ventricular systolic function, inferior vena cava measuring 2.27 cm and VExUS grading of 2 to 3. Dengue serology IgM and IgG were positive and NT-proBNP was raised at 12500 pg/ml. Instead of fluid resuscitation, diuretic and inotropes were initiated along the line of cardiogenic shock secondary to dengue cardiomyopathy. Serial normal cardiac enzymes and electrocardiogram excluded acute coronary syndrome. She was discharged well, and repeated echocardiography one-month post discharge showed normal left ventricular systolic function with no clinical signs or symptoms of heart failure.

## Introduction

Dengue fever is an arthropod-borne viral disease spread by female Aedes mosquitoes namely Aedes aegypti, Aedes albopictus, and a few other species. Dengue virus, a member of the Flaviviridae family, has five serotypes: DEN 1, 2, 3, 4, and 5 1. DEN 5 was the most recently found serotype in 2007 in Sarawak, Malaysia 1. Ongoing global warming and the expansion of urbanisation have favoured the rapid and widespread transmission of dengue fever worldwide in recent decades, with cases reported to the World Health Organization (WHO) rising from 505,430 in 2000 to 5.2 million in 2019. Dengue incidence peaked in 2023, affecting over 80 countries across all WHO regions. Since the beginning of 2023, continued transmission, along with an unexpected surge in dengue infections, has resulted in a record high of over 6.5 million illnesses and more than 7,300 dengue-related fatalities 2. 

Dengue fever can cause a wide range of clinical symptoms, from mild flu-like conditions to potentially fatal conditions with plasma leakage, bleeding, or multi-organ impairment. A dengue fever outbreak at Kandy, Central Province, Sri Lanka, from March to May 2005 that started within the premises of the General Hospital, Peradeniya – involving mainly medical students, doctors, nurses, and minor employees – showed the incidence of cardiac complications was as high as 62.5% 3. Here, patients died from shock and severe pulmonary oedema due to acute myocarditis 3. Dengue cardiomyopathy has been linked to increased duration of hospital stay^4^, and if it is not treated properly can lead to death. We report a case of dengue cardiomyopathy that was detected and treated to best effect using point of care ultrasound (POCUS) and the VExUS score. 

**Figure 1:**
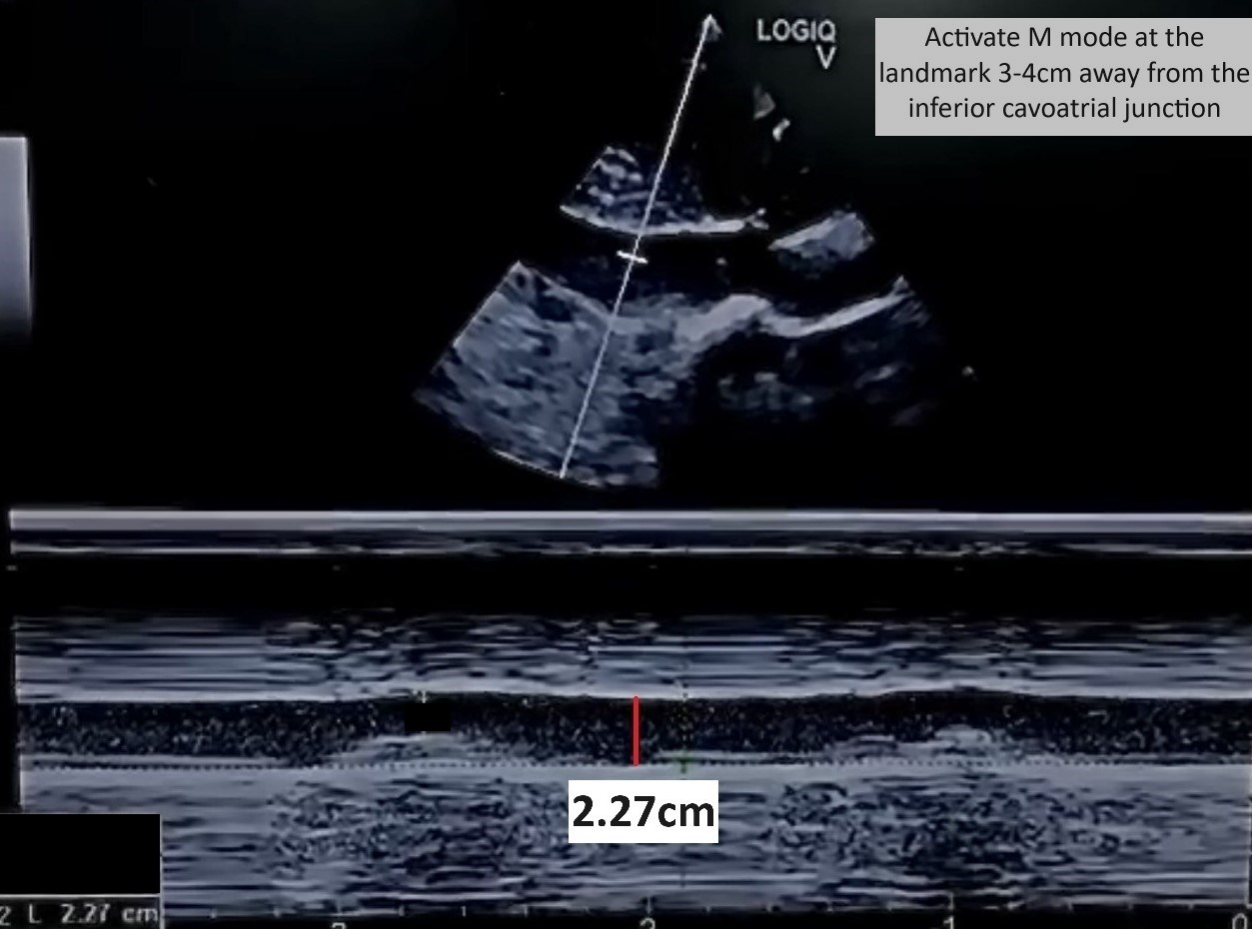
Inferior vena cava (IVC) measuring 2.27 cm

**Figure 2:**
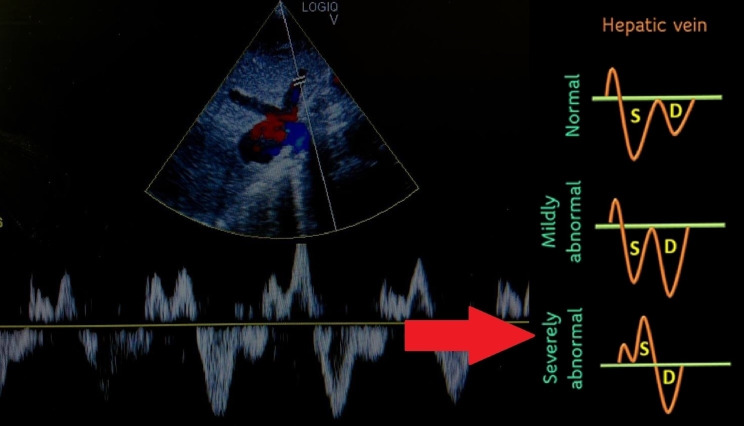
The spectral pulse wave Doppler of hepatic vein showing S reversal pattern waveform indicating severely abnormal hepatic vein.

**Figure 3:**
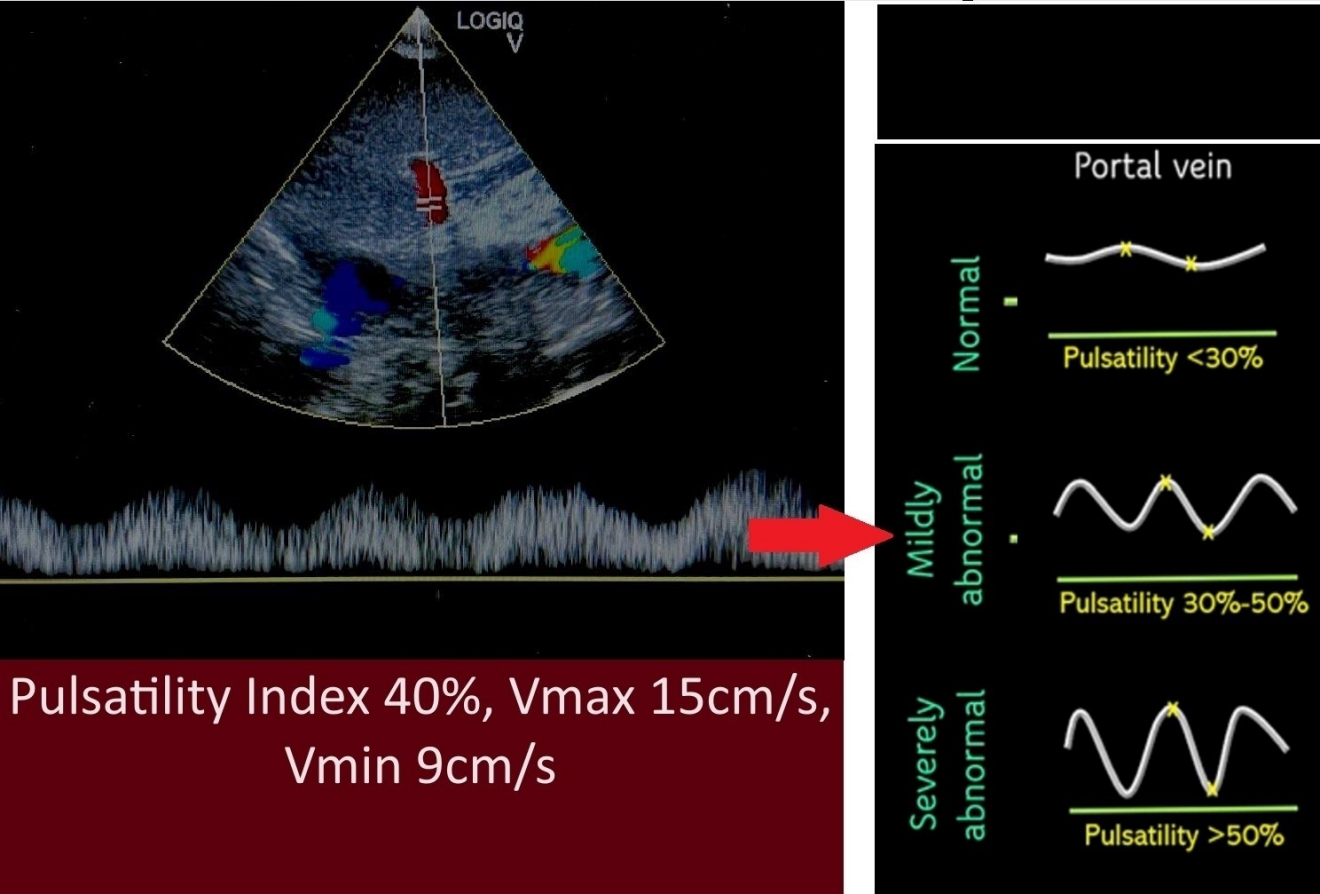
The spectral pulse wave Doppler of portal vein showing a maximum flow velocity (Vmax) of 15 cm/s with a minimum flow velocity (Vmin) of 9 cm/s with pulsatility index of 40%, indicating mildly abnormal portal vein.

**Figure 4:**
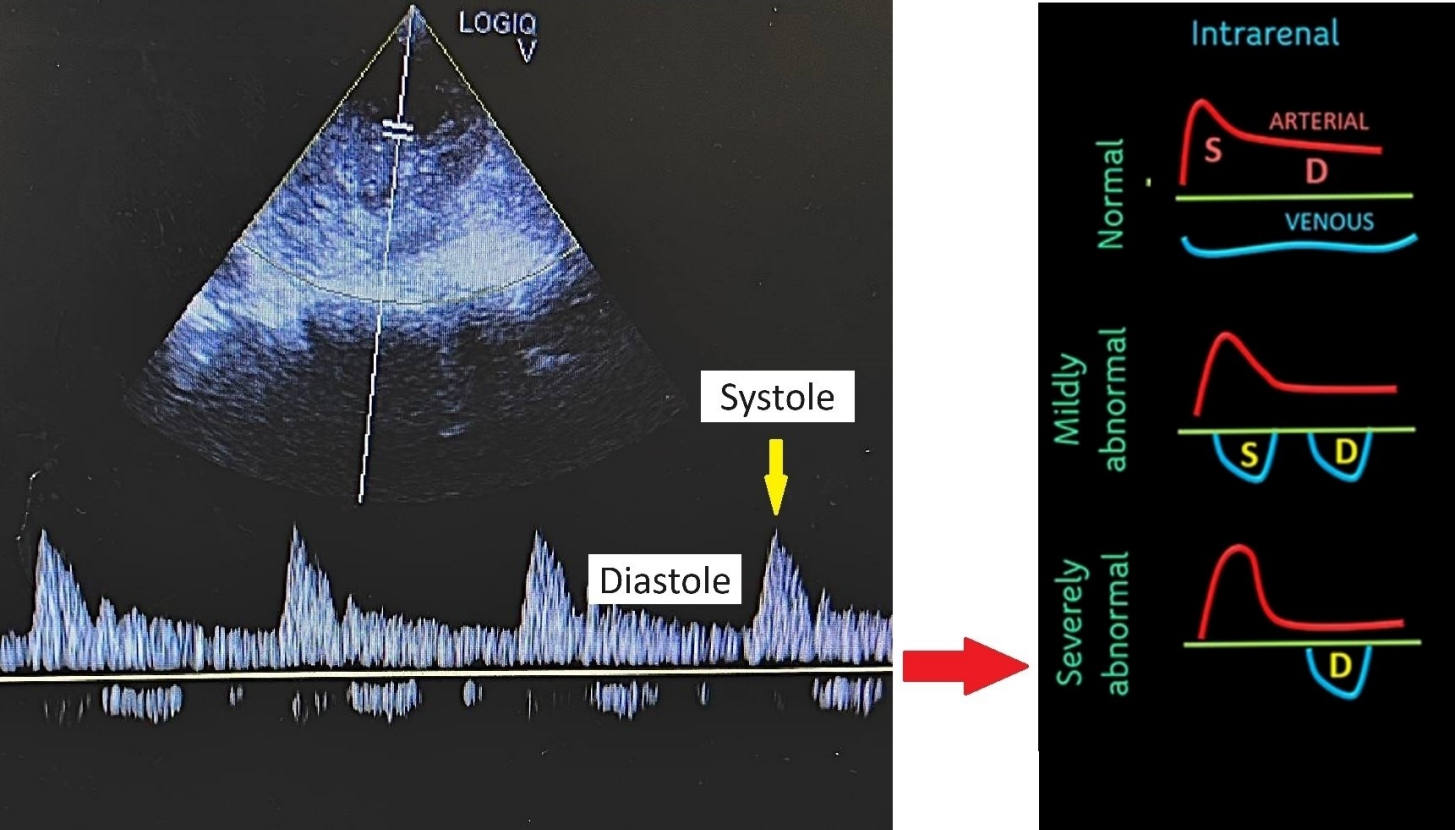
The spectral pulse wave Doppler of the intra-renal vein shows some waveforms with distinct S and D waves, while others show complete pauses during the systole phase, indicating mildly to severely abnormal intra-renal veins.

A 75-year-old lady with no known comorbidities presented to the emergency department with a 10-day history of fever, vomiting, loose stool, and poor appetite. Upon arrival, she was febrile and hypotensive, with a blood pressure (BP) reading of 88/46 mmHg. Clinically, her tongue was moist, her lungs were clear on auscultation, and there was no pedal oedema. However, the peripheral perfusion was poor, with a capillary refilling time of about 3 seconds, and limbs were cold on touch. POCUS examination was performed to determine the cause of shock. Cardiac POCUS evaluation revealed reduced left ventricle systolic function (Video S1). The measurement of the mitral valve E-point septal separation (MV EPSS) was 15 mm. Mitral annular plane systolic excursion (MAPSE) done in apical 4 chamber (A4C) view revealed septal annulus 6 mm and lateral annulus 10 mm. Besides, tricuspid annular plane systolic excursion (TAPSE) measured 20 mm. The subcostal technique was used to visualize the inferior vena cava (IVC), which was identified by the IVC's connection to the inferior cavoatrial junction and the hepatic vein's drainage into the IVC. The IVC was distended and measured 2.27 cm (Figure 1). In view of poor heart contractility and a distended IVC greater than 2 cm, we further proceeded with the VExUS scoring assessment to determine the severity of venous congestion. Spectral pulse wave (PW) Doppler was applied to the hepatic vein, portal vein, and intra-renal vein. The hepatic vein revealed an S reversal pattern (Figure 2). However, there was some limitation in interpreting the hepatic vein waveform( the absence of simultaneous electrocardiogram (ECG) tracing). As a result, we could only rely on pattern recognition to interpret the waveform rather than accurately identifying it in the diastole and systole phases. The portal vein showed a maximum flow velocity (Vmax) of 15 cm/s with a minimum flow velocity (Vmin) of 9 cm/s, resulting in a pulsatility index of 40% (Figure 3). The intra-renal vein showed some waveform with distinct S and D waves as well as some waveform showing complete pause during the systole phase (Figure 4). The spectral PW Doppler study showed a severely abnormal hepatic vein, a mildly abnormal portal vein, and a mildly to severely abnormal intra-renal vein, concluded as a VExUS grading of 2 to 3. On further bedside POCUS assessment, there was no free fluid seen in the pleural, pericardial or peritoneal cavity. Lung ultrasound performed showed B profile lung with lung ultrasound score (LUS) of 1 over the R1, R3, L1 region and LUS 2 over R2, R4, L2, L3, L4 region. 

**Figure 5:**
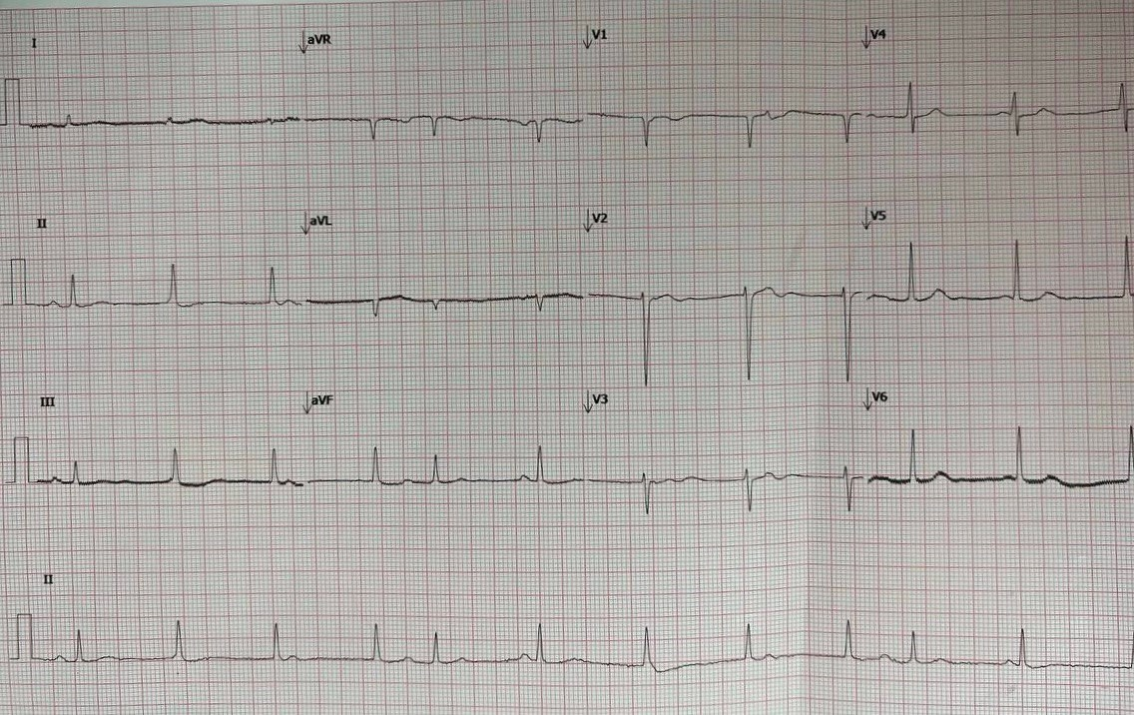
Electrocardiogram (ECG) showing atrial fibrillation.

Dengue serology revealed IgM and IgG positive. Her white cell count was 7.3 x 103/uL, and her platelet count was 53 x 103/uL. NT-proBNP was elevated at 12500 pg/mL (normal range less than 450 pg/ml). The blood gas exhibited severe metabolic acidosis with pH 7.25, bicarbonate (HCO3) 13, and base excess (BE) of -15. Lactate was elevated at 5.2 mmol/L, blood urea nitrogen at 102 mg/dl, and creatinine at 2.94 mg/dl (normal range 0.67–1.18 mg/dL). Serial creatinine kinase (CK), aspartate transaminase (AST), and lactate dehydrogenase (LDH) were normal making ischemic cardiomyopathy secondary to acute coronary syndrome less likely. In addition, the initial electrocardiogram (ECG) showed atrial fibrillation (Figure 5), with no significant ST changes seen in the subsequent serial ECG assessment. Her glucose level and fasting lipid profile were normal. The other infective screenings, namely leptospirosis serology, blood film for malaria parasites, and blood culture, were all negative. 

Diuretics and inotropes (noradrenaline and dobutamine) were initiated. Subsequently, her clinical condition significantly improved, with NT-proBNP lowered to 1350 pg/ml. The repeated bedside POCUS showed MV EPSS of 6 mm with estimated LVEF measuring 60%, IVC size 1.2 cm, and VExUS grade 0. Her metabolic acidosis resolved with her blood urea nitrogen level decreased to 54 mg/dL and her creatinine level was 1.16 mg/dL. Her repeated ECG showed sinus rhythm. She was discharged well on day eight of hospitalization. During her medical clinic follow-up one-month post discharge, her echocardiography assessment revealed normal left ventricular systolic function with no clinical signs or symptoms of heart failure. The cardiology team evaluated her situation and proposed a subsequent coronary angiography. However, she declined additional examination due to the inconvenience of long-distance travel.

**Figure 6:**
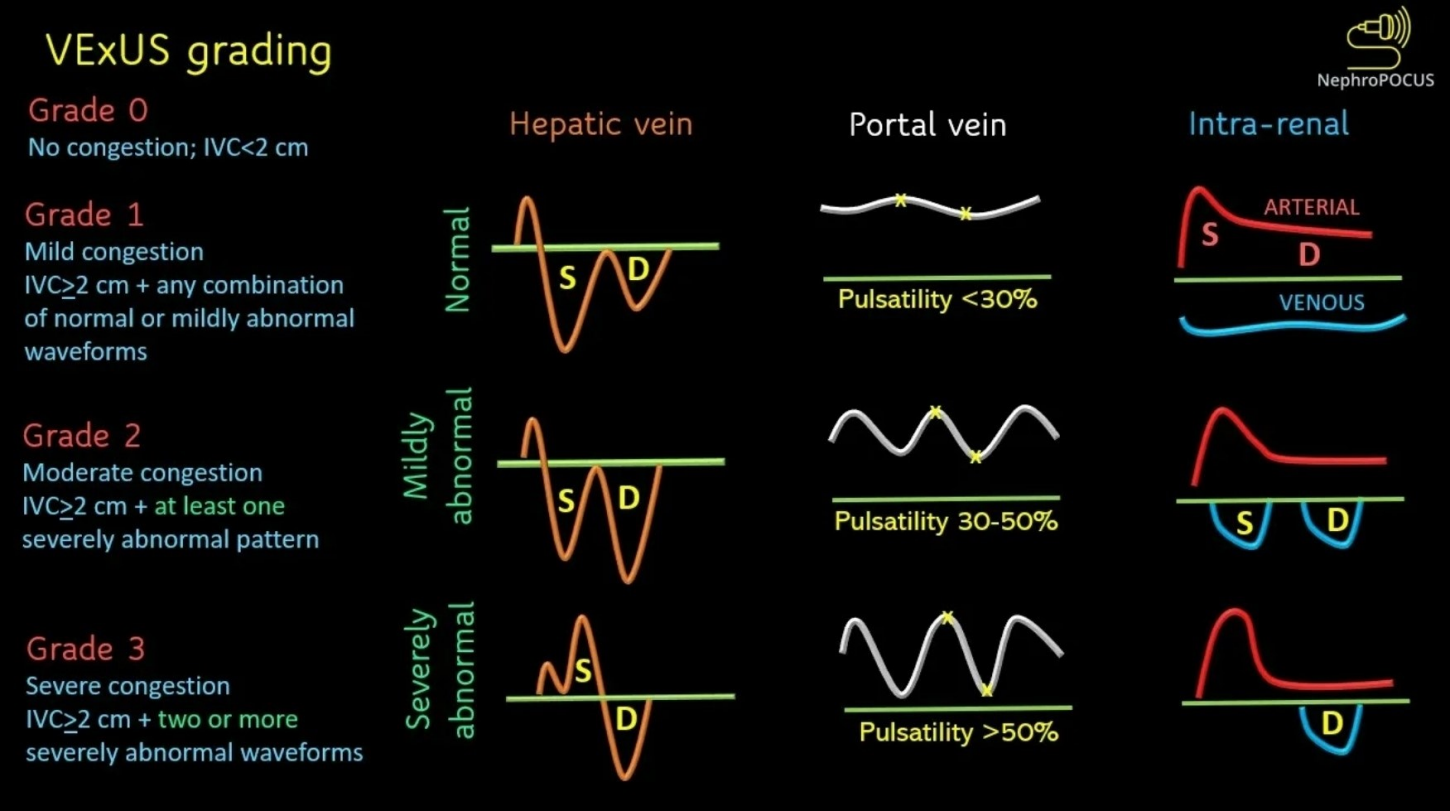
VExUS score interpretation flash card adapted from the Nephropocus website 5.

The VExUS ultrasound scoring system assesses both splanchnic and intrarenal blood circulation. It can provide useful information in evaluating the presence of venous congestion and quantifying its severity. It is a non-invasive way to identify patients who are currently experiencing fluid intolerance, and it acts as a “stop” sign to guide fluid resuscitation strategies. The interpretation of VExUS scoring is shown in Figure 6 5. In this case, if we solely relied on physical examination, we could underestimate her congestion problem as there was no pedal oedema, and her lungs were clear on auscultation. However, utilising bedside POCUS enabled us to detect the presence of the cardiomyopathy and treat the "wet and cold" heart failure in accordance with European Society of Cardiology (ESC) guidelines. Cardiac manifestations in dengue fever are not commonly reported but are probably underreported due to the challenging confirmation diagnostic test that requires a myocardial biopsy. As a result, the true incidence of dengue cardiomyopathy remains uncertain. Dengue cardiomyopathy can manifest as sudden heart failure with pulmonary oedema, cardiogenic shock, or chest discomfort mimicking an acute myocardial infarction 6. Supportive therapy remains the cornerstone of dengue cardiomyopathy care. A precise fluid resuscitation regimen may be implemented to minimise fluid overload in dengue fever cases when cardiac involvement is identified early using bedside POCUS. Application of VExUS scoring acts as an additional monitoring parameter for assessment of the response towards the management given. Integrating bedside POCUS and VExUS could lead to significant advancements in precise monitoring and management of dengue, thus reducing overall dengue fatality rates. 

## Statement of ethics approval/consent: 

Informed consent and permission for publication of the case and ultrasound images was obtained from the patient.

## Disclosure Statement 

The authors declare that they have no competing interests.

## Supplementary Material

 • Video S1
